# Matrilineal Fertility Inheritance Detected in Hunter–Gatherer Populations Using the Imbalance of Gene Genealogies

**DOI:** 10.1371/journal.pgen.0020122

**Published:** 2006-08-04

**Authors:** Michael G. B Blum, Evelyne Heyer, Olivier François, Fréderic Austerlitz

**Affiliations:** 1 Laboratoire TIMC-TIMB, Faculté de Médecine, Grenoble, France; 2 Laboratoire Ecologie, Systématique, et Evolution, Université Paris-Sud, Orsay, France; 3 Department of Human Genetics, University of Michigan, Ann Arbor, Michigan, United States of America; 4 Laboratoire d'Anthropologie Biologique, Musée de l'Homme, Paris, France; University of Chicago, United States of America

## Abstract

Fertility inheritance, a phenomenon in which an individual's number of offspring is positively correlated with his or her number of siblings, is a cultural process that can have a strong impact on genetic diversity. Until now, fertility inheritance has been detected primarily using genealogical databases. In this study, we develop a new method to infer fertility inheritance from genetic data in human populations. The method is based on the reconstruction of the gene genealogy of a sample of sequences from a given population and on the computation of the degree of imbalance in this genealogy. We show indeed that this level of imbalance increases with the level of fertility inheritance, and that other phenomena such as hidden population structure are unlikely to generate a signal of imbalance in the genealogy that would be confounded with fertility inheritance. By applying our method to mtDNA samples from 37 human populations, we show that matrilineal fertility inheritance is more frequent in hunter–gatherer populations than in food-producer populations. One possible explanation for this result is that in hunter–gatherer populations, individuals belonging to large kin networks may benefit from stronger social support and may be more likely to have a large number of offspring.

## Synopsis

Fertility inheritance is a cultural trait that may strongly decrease the genetic diversity of a population. Fertility is said to be inherited when individuals belonging to large sibships are more likely to produce numerous offspring than are individuals with few siblings. Until now, fertility inheritance in humans has been investigated using genealogical data or a combination of genetic and demographic data. In this work, the authors propose a method of detecting fertility inheritance that is based on genetic data alone. Their method relies on the reconstruction of the gene genealogy of a sample of sequences and the computation of the degree of imbalance of that genealogy. Using mitochondrial data sampled in 37 human populations, they find that fertility inheritance is more common in hunter–gatherer populations than in food-producer populations. Thus, because the human population evolved under a hunter–gatherer regime until the Neolithic transition, fertility inheritance may be one of the factors explaining the relatively low genetic diversity of the human species.

## Introduction

In some human populations, nongenetic departures from neutral evolution can occur via processes in which the number of progeny of an individual is positively correlated to the number of progeny of his/her parents [1]. In these populations, individuals whose parents had many children are more likely to have many offspring [2]. This fertility inheritance has been explained in some cases by cultural transmission [3] of a tendency for individuals to choose to have a number of offspring similar to their number of siblings. In humans, founding a large family can be seen as a cultural behavior related to family structure, and it has been shown that cultural traits related to the family are mainly transmitted by the parents [4,5].

Some effects of fertility inheritance on genetic diversity have already been studied. For instance, Nei and Murata [6] showed that fertility inheritance strongly reduces the effective sizes of populations. Austerlitz and Heyer [7] showed that it may explain the high frequencies of some genetic diseases in several populations. They also showed that because it increases the level of association between a disease locus and closely linked marker genes, fertility inheritance has consequences for the mapping of loci involved in genetic disorders [8].

Until now, this intergenerational correlation of offspring size has been detected mainly using genealogical databases [7]. For many populations, however, such databases are not available. Recently, Austerlitz et al. [9] developed an indirect way to detect fertility inheritance from genetic and demographic data. Their method uses haplotypic data to estimate jointly the age of a given mutant allele and the growth rate of the number of carriers of this allele since the time of its appearance. When the estimated growth rate of the number of carriers is higher than the known population growth rate for several independent mutations, it is likely that a demographic phenomenon such as fertility transmission is occurring in the population. This effect has been detected in two populations: Bulgarian gypsies (Vlax) and French Canadians (Saguenay-Lac Saint Jean) [9].

In this study, we aim to detect a generational correlation in fertility from genetic data, using coalescent methods. Detecting fertility inheritance differs from detecting natural selection, in part because fertility inheritance affects the whole genome whereas the effects of natural selection on a specific variant are likely to be restricted to loci located near the selected allele. It is well-known that population demography has an effect on the shape of gene genealogies [10]. For instance, genealogies in a growing population will tend to be more star-like [11–13]. Similarly, a departure from the neutral coalescent is found when fertility is inherited. In particular, Sibert et al. [14] showed that fertility transmission causes a decrease in coalescence times. This reduction is higher in the branches near the MRCA, giving a star-like shape to the coalescent tree. However, since a similar shape is also expected in an expanding population, inferring only the lengths of the branches of the tree is not enough to infer fertility inheritance. Thus, Sibert et al. [14] identified a specific signature of fertility transmission: it increases the level of imbalance of coalescent trees.

Although it has not been of interest in coalescent theory until now, the balance and imbalance of trees have been widely studied in systematic biology to test hypotheses about macroevolutionary processes [15]. The imbalance of a tree is defined as the average level of imbalance of its nodes, assuming that a given node is completely balanced if it splits the sample into two subsamples of equal size, and that its imbalance increases with the difference in the sizes of the two subsamples. Several measures computing the balance of a whole tree [16] as well as measures for individual nodes have been proposed. In this paper, we show how one of these whole-tree balance measures, the mean *I′* [17], can be used with a sample of DNA sequences as a method for detecting fertility correlation in a population (see Materials and Methods). Our method consists in reconstructing the genealogical tree of these sequences using phylogenetic methods, and in computing the mean *I′* value of this tree and its level of statistical significance under the null hypothesis that no fertility correlation exists. Under the null hypothesis, the mean *I′* is expected to equal 1/2. A value of the mean *I′* significantly larger than 1/2 is considered as evidence of fertility transmission.

To assess the validity of our method, we first perform a power study, based on simulated replicates, to determine the impact of the level of fertility transmission on the mean *I′* computed from coalescent genealogies. Second, we test the robustness of our method under different demographic scenarios. Third, we also investigate the influence of the phylogenetic method used. Finally, we apply our test to mtDNA samples from 37 human populations and study whether fertility transmission is more frequently detected in traditional hunter–gatherer populations (HGPs) or in food-producer populations (FPPs).

## Results

### Power of Tests Based on Tree Imbalance

Simulations were performed using the model of Sibert et al. [14], which assumes that the propensity to reproduce for an individual is proportional to *,* where *s_i_* is the sibship size of the individual *i*. Here *α* denotes the intensity of fertility inheritance and ranges from zero to two. Strong fertility inheritance with *α* > 1 is clearly detected by an increase in mean *I′* (Figure 1). Mean *I′* increases monotonically and continuously from *α* = 0 to *α* = 2. Thus, because this statistic gives the same weight to all nodes, the number of unbalanced nodes increases continuously with *α*. Therefore, the imbalance signal is not confined to the basal nodes only.

**Figure 1:**
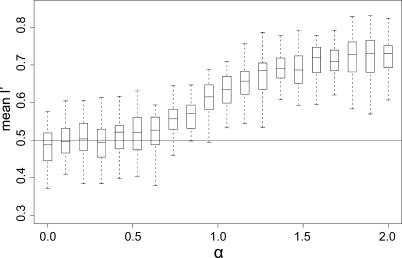
The Effect of Fertility Inheritance on the Balance Measure Mean *I*′ For each value of the fertility inheritance parameter *α,* 100 coalescent trees with 100 individuals were simulated. The extremities of the boxes correspond to the first and the third quartiles. The horizontal line in each box represents the median, and the whiskers extend to the most extreme data points. The horizontal line indicates 0.5, the expected value of mean *I*′ under the neutral coalescent.

To investigate the power of the test, simulations were performed for different values of the sample size *n* and the intensity of fertility inheritance *α* (Table 1). The test is unable to detect fertility inheritance for *α* < 1, even when *n* = 100. As *α* increases, the transition in power to detect fertility inheritance occurs surprisingly rapidly. For *α* ≥ 1, the test performs well even when the sample sizes are small (power = 0.68 for *n* = 20 and *α* = 1.33). For large sample sizes and *α* > 1, the power of the test is close to 1.

**Table 1:**
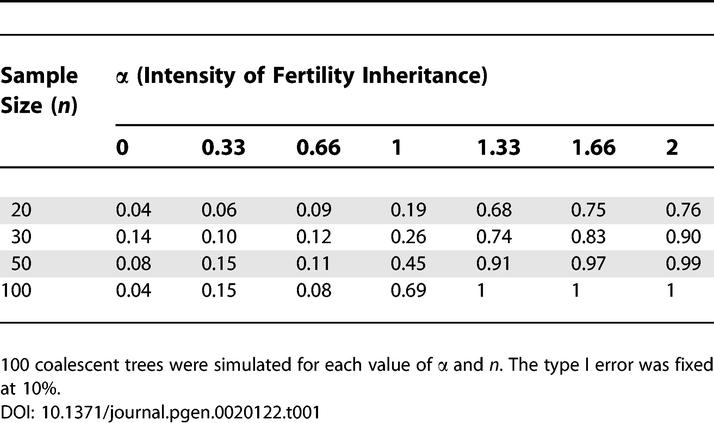
The Power of the Mean *I′* Test to Detect Tree Imbalance for Several Values of the Fertility Inheritance Parameter *α*

### Robustness of the Method

The above results assume an isolated population of constant size, with constant intensity of fertility inheritance over time. However, these assumptions are often violated in real cases. Therefore, we performed simulations to determine if fertility correlation can be detected in expanding populations or in populations where fertility is transmitted only during short periods of time. Moreover, we performed simulations without fertility transmission in geographically structured populations to determine whether population structure could yield a spurious signal of fertility transmission.

#### Population expansion.

We assumed that the population remained at a constant size until ten or 100 generations before the present, when it started to grow geometrically until the present (*N*(*t* + 1) = *kN*(*t*)). We assumed growth rates (*k*) of 1.01 and 1.03 per generation for the population that experienced an expansion 100 generations before the present, and of 1.2 and 1.4 for the population that experienced an expansion ten generations before the present.

We found that the gene genealogies simulated in the models of population expansion exhibited almost exactly the same imbalance pattern as in the constant population size model (see Figure 2). As in the constant population size model, fertility inheritance could be detected for intensities *α* greater than one. Moreover, the value of the growth rate had very little influence on the power of the test.

**Figure 2:**
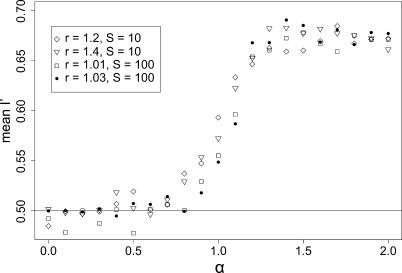
The Effect of Population Expansion and Fertility Inheritance on the Balance Measure Mean *I*′ For each value of the fertility inheritance parameter *α* and each scenario of population expansion, 100 coalescent trees with 100 individuals were simulated. Each point corresponds to the mean value of the mean *I*′ for the 100 replicates. The parameter *r* denotes the geometric rate of the population expansion and *S* denotes the time, measured in generations, since the beginning of the expansion.

#### Fertility inheritance in a limited period of time.

Assuming strong fertility inheritance during a large number of generations may be unrealistic. For instance, in the French Canadian population studied by Austerlitz and Heyer [7], fertility inheritance was observed in the genealogical databases over a period of approximately ten generations. To take the possibility of a restricted duration of fertility inheritance into account, we simulated populations that experienced fertility inheritance only during a period of time that started *T* generations before the present and that lasted until *T* − *τ* generations before the present. *T* ranged from zero to 60 generations, and *τ,* the length of time over which fertility inheritance occurred, was set to either five or ten generations. When fertility correlation occurred in the most recent generations, the pattern of imbalance remained very similar to the pattern shown in Figure 1. In contrast, imbalance is harder to detect in a population that experienced *τ* = 10 generations of fertility inheritance *T* = 110 generations ago even if the level of fertility inheritance was substantial. The transition between these two extreme scenarios is continuous. The threshold value of *α* above which fertility inheritance can be detected increases as *τ* decreases and *T* increases (Figure 3). We observed in particular that strong fertility inheritance may leave a substantial fingerprint even when it happens over a period of only five generations. However, the signal of imbalance disappeared if fertility inheritance occurred only in the distant past. In a scenario where fertility was inherited over a period of 500 generations until 500 generations before the present (corresponding roughly to 10,000 years BP), we were unable to detect tree imbalance.

**Figure 3:**
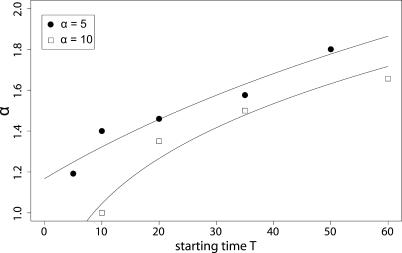
The Effect of the Starting Time *T* of the Fertility Inheritance Process on the *α* Value above Which It Is Possible to Detect Imbalanced Trees Tree imbalance was detected when more than 90% of the simulated trees were more unbalanced than expected under the neutral model (type I error = 10%). The starting time is measured backward in time. The parameter *τ* denotes the length of time, measured in generations, over which fertility inheritance occurred. The solid lines have been obtained using a linear regression between log *α* and log *T.*

#### Spatial structure.

We simulated three different models of population structure: a two-island model, a model of population merging, and a model of spatial range expansion (see [18]). In the two-island model, with conservative migration (*N*_1_ *m*_12_ = *N*_2_ *m*_21_), the balance of the genealogies that link individuals from the same population does not differ to a great extent from the neutral expectation when the sampling is made in one population only (Table 2). We observed unbalanced trees only when the two population sizes differed by a factor of 100 and when the migration rate was rather high (*N*_1_ *m*_12_ = 10). The proportion of unbalanced trees increased when the sampled individuals did not come from the same population, especially when the difference in population sizes was large.

**Table 2:**
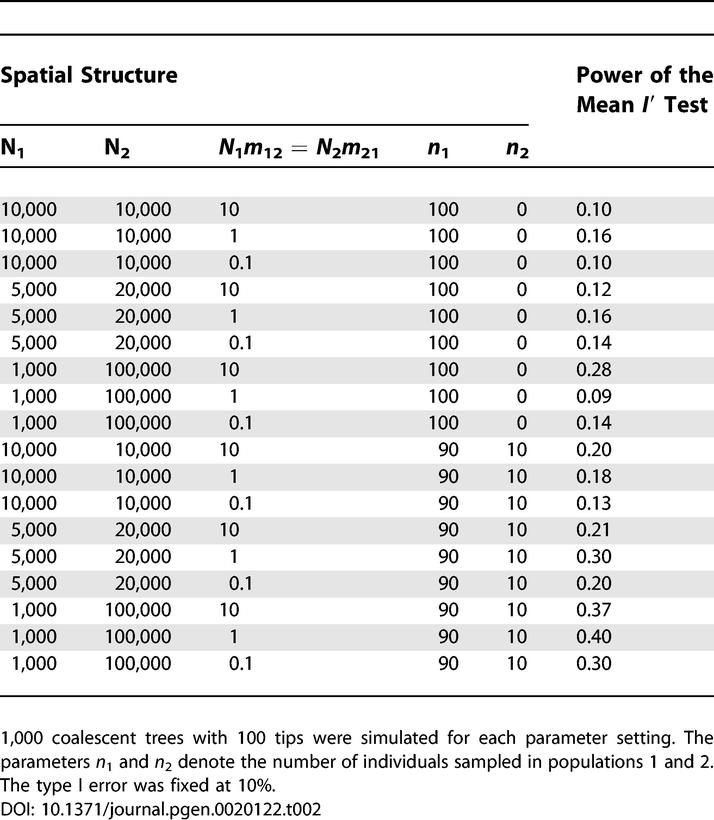
The Power of the Mean *I′* Test to Detect Tree Imbalance under a Two-Island Model with Conservative Migration

The results for nonconservative migration (*N*_1_ *m*_12_ ≠ *N*_2_ *m*_21_) are similar to the results for conservative migration (Table 3). Once again, we did not observe many unbalanced trees, except when the two population sizes differed by a factor of 100. The scenario of one population experiencing a population expansion and exchanging migrants with a small population of constant size produced some unbalanced trees (Table 3) when the sampling was done in both populations. The other models of spatial structure that we considered, namely a scenario of population merging and a scenario of range expansion, did not generate substantial tree imbalance (Tables 4 and 5).

**Table 3:**
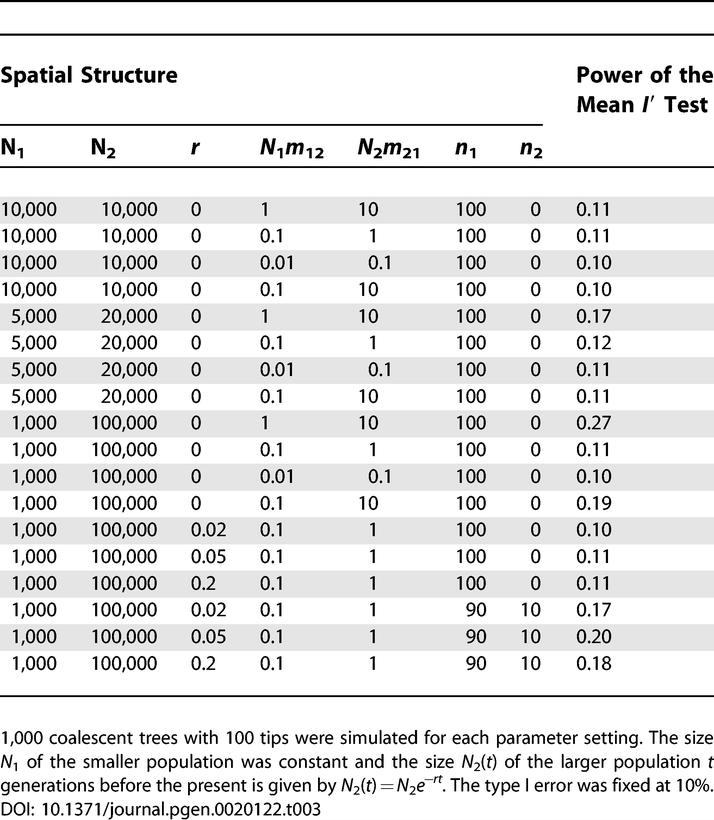
The Power of the Mean *I′* Test to Detect Tree Imbalance under a Two-Island Model with Nonconservative Migration

**Table 4:**
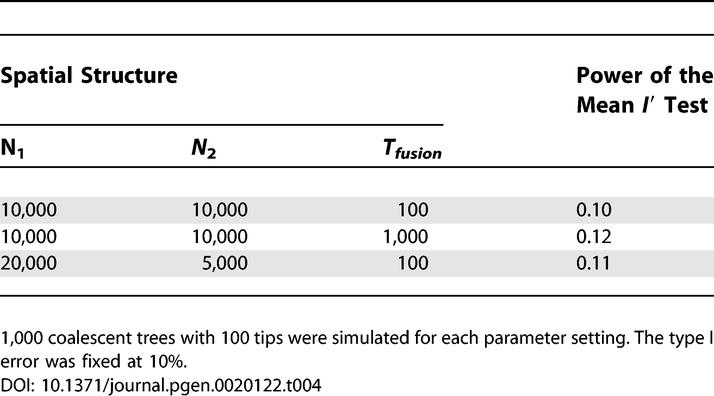
The Power of the Mean *I′* Test to Detect Tree Imbalance under a Model of Population Merging

**Table 5:**
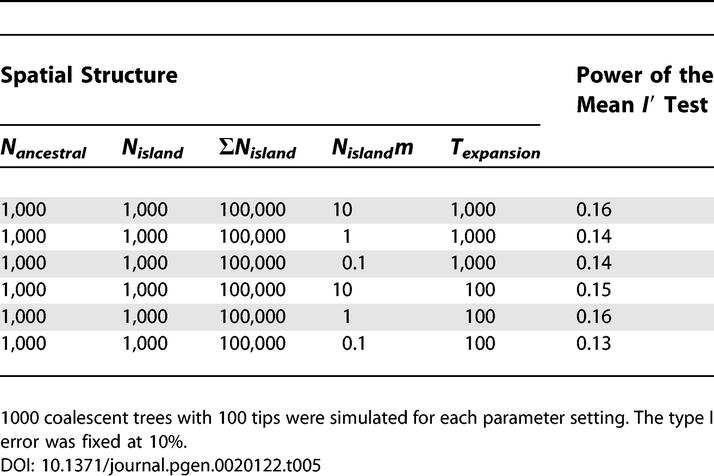
The Power of the Mean *I′* Test to Detect Tree Imbalance under a Model of Range Expansion

In brief, spatial structure may generate tree balance but not to the same extent as does fertility inheritance. In addition, it occurs mostly when individuals are sampled in different populations.

#### The effect of tree reconstruction.

Because gene genealogies are unknown in practice and are only reconstructed from DNA datasets, the effect of tree reconstruction on imbalance needs to be investigated. For this reason, we simulated genetic data using two different mutation rates and we inferred the genealogical trees of the sequences using the reconstruction methods UPGMA and maximum likelihood, as implemented in PHYML [19]. For both mutation rates, the genetic diversity of the population is shown in Table 6. As was also shown by Sibert et al. [14], diversity decreases when fertility inheritance increases, especially for the lower rate, making it difficult in that case to reconstruct gene genealogies under high fertility correlation intensities. In the following, only the larger mutation rate is considered. We assumed either homogeneous or heterogeneous mutation rates along the sequences. In the latter case, the mutation rates of each site were drawn according to a gamma distribution with shape parameter 0.26, in accordance with a previous estimate from human HV1 sequences [20].

**Table 6:**
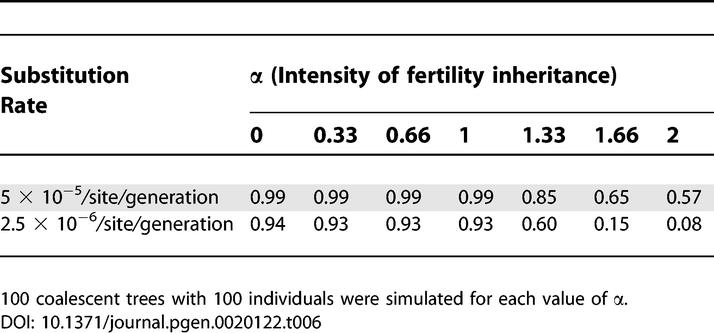
The Effect of Fertility Inheritance on Heterozygosity

The power of imbalance tests on reconstructed genealogies is shown in Table 7. Thirteen percent of the PHYML trees and 38% of the UPGMA trees were more unbalanced than predicted by the neutral coalescent for homogenous mutation rates, and *α* = 0. Since the expected number of rejected replicates is 10%, it means that gene genealogies reconstructed with the UPGMA method are often more unbalanced than expected under the neutral coalescent, while the PHYML method does not produce an elevation in imbalance. The power to detect unbalanced trees increases with *α* for both methods of tree reconstruction. For *α* > 1, all UPGMA topologies and most PHYML topologies are more unbalanced than predicted by the neutral coalescent. Detection of fertility inheritance on PHYML-reconstructed trees is possible when mutation rates are heterogenous along the sequence. However, the level of spurious detection of fertility inheritance using PHYML-reconstructed trees may be increased when there is heterogeneity in mutation rates.

**Table 7:**
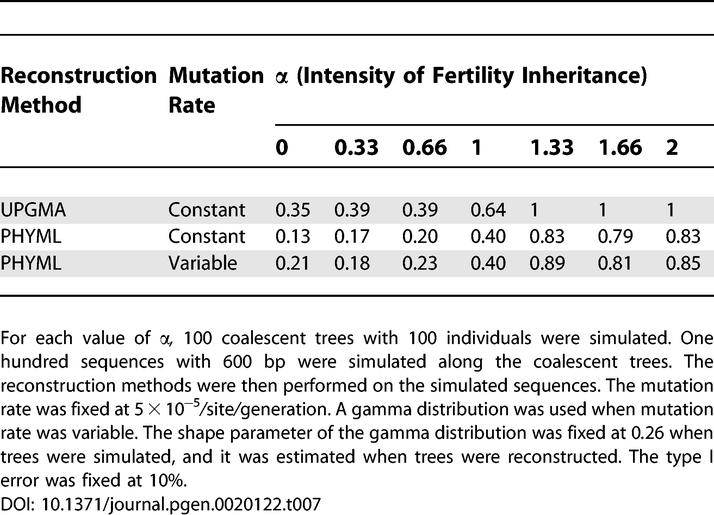
The Power of the Mean *I′* Test to Detect Tree Imbalance Using Reconstructed Gene Genealogies

### Experimental Data

We applied our method to mitochondrial datasets from the database MOUSE [21], a compilation of mtDNA from hypervariable regions I and II of the D-loop. Because MOUSE contains a fairly small number of mtDNA samples from HGPs, we also considered mtDNA samples from one additional Asiatic HGP [22] and two African HGPs [23,24]. Since mtDNA is maternally inherited, mtDNA tree balance reflects fertility transmission from mother to daughter. The balance of the reconstructed trees for human mtDNA is shown in Table 8 for HGPs and in Table 9 for FPPs. The number of resolved nodes (i.e., the nodes that give rise to two lineages only) is highly variable (mean = 12.67 and s.d. = 10.04), positively correlated to the number of sequences available (*R* ^2^ = 0.76) and significantly lower in HGPs (*p* = 0.001 for a one-sided Wilcoxon rank test). The heterozygosity is significantly lower in HGPs than in FPPs (*p* = 0.0002 for a one-sided Wilcoxon rank test) and is correlated with the tree balance index (*p* = 0.02 for a Spearman rank test).

**Table 8:**
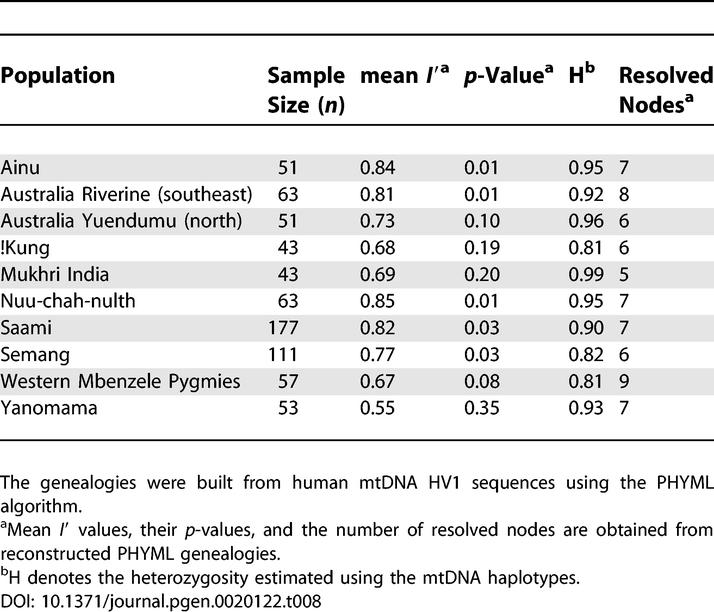
mtDNA Genealogy Imbalance for HGPs

**Table 9:**
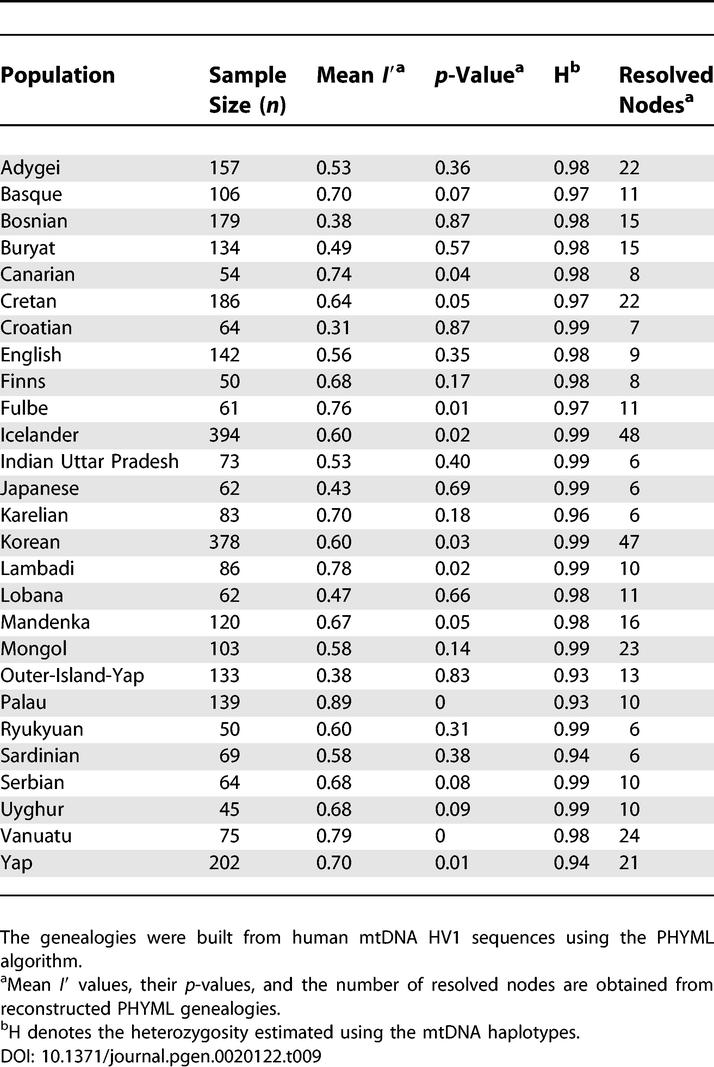
mtDNA Genealogy Imbalance for FPPs

Fertility correlation appeared to be much more frequent in HGPs. Indeed, the mean of the imbalance index is significantly larger in HGPs (*p* = 0.005 for a one-sided Wilcoxon rank test) equaling 0.74 (s.d. = 0.10) in HGPs and 0.60 (s.d. = 0.14) in FPPs. Moreover, only ten of 27 FPPs showed a significant mean *I*′ (*p* ≤ 0.05), while the mean was significant for five of ten HGPs.

## Discussion

This study illustrates that tree imbalance is a convenient way of detecting fertility inheritance. Although many statistics have been proposed to capture tree balance [16], the statistic that we used here (mean *I*′*,* [17]) seems to be well-adapted to gene trees because it has been designed to deal with partially resolved trees. Moreover, this statistic is not strongly affected by variations in population size and is not too sensitive to hidden geographical structure. Indeed, unbalanced trees are only produced when this geographical structure is strong, for example when a structured population consists of two groups with very different sizes that exchange relatively few migrants (1 ≤ *Nm* ≤ 10). Thus, it is possible that some imbalanced trees may have been produced by spatial structure especially for HGPs that are exchanging migrants with their neighbors. If estimates of the degree of contact between HGPs and FPPs were available for all of the populations we have studied, then the correlation between the extent of contact and the degree of tree imbalance could potentially be investigated.

The tree balance statistic is, moreover, slightly affected by the sampling procedure. The introduction of a substantial number of outliers from a large population into the sample may indeed increase the level of imbalance. However, it seems unlikely that the number of outliers is large in the HGP samples where fertility inheritance has been detected, since when FPPs and HGPs coexist in the same areas, more migration is expected from HGPs to FPPs than in the other direction.

It should be stressed that our method detects fertility inheritance only when its intensity is high, namely *α* > 1, which corresponds approximately to a correlation in fertility between parents and offspring of 0.2 [14]. However, if this high rate of fertility correlation has been reached during just a few generations, we have shown that it still strongly modifies tree balance and can thus be detected, even after 50 generations (100 in some extreme cases). Fertility correlations of about 0.2 have been observed in human populations. For instance, Austerlitz and Heyer [7] obtained values between 0.161 and 0.34 for the Saguenay-Lac Saint-Jean population. Similarly, Draper and Hames [25] found a correlation of 0.255 for the !Kung from Botswana. In this population, the correlation was larger for males (0.447) than for females (0.076), suggesting that Y-chromosomal data may be used to determine if this larger amount of patrilineal fertility inheritance applies to human populations more generally. However, Helgason et al. [26] found a greater level of fertility inheritance for matrilines than for patrilines in Icelandic genealogical data. Their evidence of matrilineal fertility inheritance in the Icelandic population is supported by the level of tree imbalance that we have observed.

Another important point is that since the genealogical tree must be inferred from genetic data attention has to be paid to the quality of this reconstruction. Our method works properly only if the samples are polymorphic enough to enable phylogenetic methods to resolve a substantial fraction of the nodes of the genealogy. Indeed, simulations showed that the power of the method is strongly decreased when there are fewer than four resolved nodes (unpublished data). Since fertility inheritance reduces genetic diversity, its detection could be hindered in some cases by a lack of polymorphism. In particular, this can be an issue for HGPs that are known to have a strongly reduced level of genetic diversity [27]. Thus, the method will clearly perform better on rapidly mutating sequences such as HV1, or on sequences long enough to have accumulated a sufficient number of polymorphic sites. In that context, the maximum likelihood method (PHYML) is preferable to UPGMA. Indeed, the UPGMA method tends to yield excessively unbalanced trees, and so its use would lead to inappropriate rejections of the neutral coalescent. It has been observed by Huelsenbeck and Kirkpatrick [28] that the ability of a method to infer the shape of a phylogeny is correlated to the accuracy of the method, suggesting that PHYML performs better than UPGMA at inferring phylogenies.

Our results are based on a sizable set of HGPs and FPPs, and the difference in the mean value of *I*′ between these two groups of populations is highly significant. Thus, we may conclude that fertility transmission appears to be more common in HGPs than in FPPs. This result could provide a partial explanation for the lower genetic diversity found in these populations, although other factors, such as the recent bottlenecks that may have occurred in HGPs after the Neolithic transition, may also explain this reduced genetic diversity [29]. The differences between mtDNA diversity in HGPs and FPPs have also been explained by differences in the rate of migration that may occur between neighboring populations after a spatial expansion [30]. However, the range-expansion model that we have considered [18] does not produce tree imbalance even when migration rates are low, suggesting that a range-expansion scenario does not explain all the features of the genetic diversity observed in HGPs. In any case, the high mean *I*′ value for HGPs advocates for the importance of fertility transmission in these populations. Note that we cannot rule out the possibility that fertility transmission is linked to a natural selection process, since we focused here on a single mitochondrial gene, the HV1 sequence. However, to have such an impact on tree topologies, this process would need to have been strong and recent, and it would be rather surprising if this process had affected only HGPs.

On the other hand, there are several cultural factors that may explain strong fertility inheritance in HGPs. First, in HGPs social organization is often based on cooperative kin networks. Thus, individuals from a large sibship may receive more help in childrearing and may thus have the capacity to support more children themselves. This argument has been proposed for explaining the correlation between sibship size and fertility in the Ache living in Paraguay [31] and the !Kung in Botswana [25]. Moreover, in HGPs the propensity to reproduce may also depend on the size of the lineage. For instance, in the Yanomama population, Chagnon [32] has shown that men belonging to large lineages were able to find wives for their numerous sons because they have many female relatives to exchange. The fertility correlation detected in HGPs may be partially due to this type of lineage dependency.

It is interesting that we did not find much fertility transmission in FPPs. Indeed, as FPPs are often socially stratified [33], with the social status being transmitted from one generation to the next, some fertility inheritance could have been expected. Our results here nevertheless indicate that inheritance of status, even if common in FPPs, does not leave a strong signal in the shape of mitochondrial gene trees. Note, however, that the separation between the two types of populations, FPPs and HGPs, may not necessarily reflect differences in the cultural traits relevant to production of fertility inheritance. Some traditional FPPs from Melanesia and Micronesia (Palau, Vanuatu, Yap), for instance, leave a signal of fertility inheritance in genealogical shape that may be explained by the cultural traits invoked for HGPs. A comparison of fertility inheritance between groups stratified by other variables would potentially be quite informative about the cultural determinants of fertility inheritance.

In conclusion, we have devised a test that detects fertility inheritance from the imbalance of reconstructed gene genealogies. Our study of HV1 mitochondrial sequences shows that fertility inheritance is much stronger for females in HGPs than in FPPs, perhaps due to cultural factors such as cultural kin networks. On a global scale, the whole human population has lived as hunter-gatherers until very recently. Thus, fertility transmission may have been quite common in our species before the Neolithic period, and it may be one of the factors explaining the low diversity of our species and the low estimated time to the common ancestor of all mitochondria. Moreover, fertility transmission could be higher for males, as polygamy has been shown to be socially inherited in some populations [32]. Data accumulating on the Y chromosome will allow us in future work to determine if similar processes have occurred along patrilines.

## Materials and Methods

### Tree balance measures.

We focus here on the imbalance of the trees, ignoring branch lengths. Most of the statistics capturing tree imbalance [16] assume that trees are fully resolved. This assumption will often not be fulfilled—for example, if several sampled individuals carry exactly the same sequence (e.g., mtDNA in HGPs [29]), or if the gene genealogies cannot be reconstructed entirely. However, the fact that a branch leads to several individuals rather than to one is informative about imbalance. Fusco and Cronk's [34] method for detecting imbalance, as modified by Purvis et al. [35], has been devised to accommodate incompletely resolved trees. In this method, only the subtrees with more than three tips (i.e., with more than one topology for a given tree size) are considered. For each node giving rise to such a subtree, this method computes

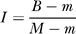
where *B* is the size of the larger daughter clade, 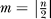 is the minimum value for *B,* and *M* = *n* − 1 is the maximum value for *B*. To devise a statistic whose expected value is independent of *n,* Purvis et al. [35] proposed the following modification

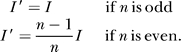
For each node, the expected value of *I*′ is 0.5 for a neutral coalescent tree [35]. To compute a summary statistic for the whole tree, Agapow and Purvis [17] considered the mean of *I*′ across all nodes for which the phylogeny is resolved. If the mean *I*′ is greater than 0.5, the tree is more unbalanced than expected for a neutral coalescent tree. Since the statistic is normalized, trees of different sizes can be compared using mean *I*′. It is worth noticing that all resolved nodes contribute equally to mean *I*′*,* and since there are more nodes near the tips of the tree, this statistic is mostly influenced by nodes near the tips [17].

To assess whether mean *I*′ is significantly higher than 0.5, the expected value for a neutral coalescent tree, we adopted the same randomization procedure as used by Agapow and Purvis [17]. For each tree, 5,000 randomizations were performed. One randomization consists of replacing, for all the nodes of the tree, *I*′ by 1 − *I*′ with probability 1/2, and recomputing the value of mean *I*′ for the whole tree. The *p*-value for the neutral coalescent hypothesis is the fraction of means computed on randomized trees that are greater than or equal to the observed mean. As stressed by Agapow and Purvis [17], the randomization test can be applied to incompletely resolved trees. All statistical computations were performed with the free software R, using the phylogenetic tree analysis packages APE [36] and apTreeshape [37].

### Simulation study: Model for the coalescent with fertility correlation.

We used a simulation approach that allowed us to compare Kingman's [38] coalescent, which corresponds to the classical Wright–Fisher model without fertility transmission, with cases where fertility is inherited. While several models have studied fertility correlation (e.g., [7,39,40]), Sibert et al.'s [14] model was the most straightforward to use here, since it is the only one that is a direct extension of the Wright–Fisher model.

This Wright–Fisher model describes the evolution of a haploid population of constant size *N,* where at each generation *t,* the parent of each individual is drawn at random with replacement among the individuals from generation *t* − 1. Each parent has the same probability to be drawn. On the other hand, in the model of Sibert et al. [14], the probability of a given individual *i* to be drawn as a parent depends on its own parent's progeny size—in other words its sibship size, denoted *s_i_*. The probability for *i* to be chosen as a parent is set to 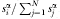 , where *α* is the intensity of fertility inheritance (*α* ≥ 0). The range of values of *α* that we consider is between zero and two. A value of *α* = 0 corresponds to the classical Wright–Fisher model without fertility inheritance. When *α* = 2, the intensity of fertility inheritance is very strong: an individual with two siblings for instance will have a propensity to reproduce that is nine times larger than that of an only child.

### Simulation study: Impact of fertility transmission on tree imbalance.

Populations of constant size *N* = 5,000 were simulated according to the fertility inheritance model described above. Setting *α* to a given value, we simulated the population for a large number of generations and sampled at random *n* individuals from the most recent generation. Since we stored all relevant population lineages, we were able to trace back the complete genealogy of the sampled individuals. We performed 100 replicate simulations for 20 values of *α* ranging from zero and two and computed in each case the mean and standard deviation of the observed mean *I*′. This allowed us also to determine the threshold value of *α* above which *I*′ is expected to diverge significantly from 0.5 and for which fertility transmission can be detected.

### Simulation study: Robustness of the method.

For the scenario of population expansion and the scenario where fertility inheritance is experienced during few generations, we performed repeated simulations to compute mean *I*′ for the same range of values of *α* as above, to determine whether or not the threshold value of *α* above which fertility transmission can be detected by *I*′ is affected. For the population structure case, we performed simulations without fertility transmission to determine whether spatial structure could yield a spurious signal of fertility transmission.

### 1. Spatial structure.

We simulated three different models of population structure.

*Two-island model with conservative and nonconservative migration*. The parameters *N*_1_ and *N*_2_ denote the effective sizes of the two populations, *m_ij_,* the migration rate (viewed backward) from population *i* to population *j,* and *n_i_,* the number of sampled individuals in population *i*. Migration is said to be conservative when the effective numbers of migrants, *N*_1_ *m*_12_ and *N*_2_ *m*_21_, are the same in both directions. We also consider the case where the population size of one of the populations is not constant but grows exponentially at rate *r*. The population size of this population at time *t* in the past is given by *N*(*t*) = *N*(0)*e* ^−*rt*^, where *t* is measured in generations.

*Model of population merging*. Two separated populations (of effective size *N*_1_ and *N*_2_) merged *T_fusion_* generations ago and gave rise to a new population.

*Range-expansion model of Excoffier [18]*. This model assumes an ancestral population of effective size *N_ancestral_,* from which an instantaneous expansion occurred *T_expansion_* generations ago. During this instantaneous expansion, all individuals from the ancestral population colonize a large number of islands. After the expansion, each island has an effective size of *N_island_* and exchanges migrants with all the other islands, with the same migration rate (*m*). The sum of the effective sizes over all the islands is denoted ∑*N_island_,* and all sampled individuals are assumed to come from the same island.

For each case, coalescent trees with 100 individuals were simulated with the software SIMCOAL [41].

### 2. The effect of tree reconstruction.

We simulated coalescent trees with 100 individuals for several values of *α*. Fertility inheritance was only experienced during ten generations beginning 20 generations ago. DNA sequences were generated along the coalescent trees using Seq-Gen [42], according to the Hasegawa-Kishino-Yano (HKY85) substitution model, assuming equal base frequencies and a transition–transversion ratio of four [43]. The mutation rate was set either at 5 × 10^−5^/site/generation, as estimated for human mtDNA from pedigree studies [44], or at 2.5 × 10^−6^/site/generation, as estimated from the divergence time between humans and chimps [45]. The sequence length was set at 600 bp. To take into account the fact that mutations do not evolve at the same rate at different positions on the HV1 sequence, we performed an extra set of simulations where the mutation rate for each nucleotide was drawn according to a gamma distribution with a shape parameter equal to 0.26 [20]. The coalescent trees of the simulated sequences were reconstructed either with UPGMA or a maximum-likelihood method. UPGMA trees were built with PHYLIP [46] from a ML distance matrix estimated using the HKY85 model (the transition ratio was estimated). The maximum-likelihood reconstructions were performed with PHYML [19] using the BIONJ tree [47] as the starting tree, and using the HKY85 model with an estimated transition ratio. When the mutation rate was heterogeneous along the sequence, the shape parameter of the gamma distribution was estimated by PHYML and the number of substitution rate categories was fixed at four. ML trees were rooted using a simulated outgroup. We performed 100 replicates for each value of *α,* and in each case we reconstructed the coalescent tree with both methods. We discarded reconstructed trees with fewer than four resolved nodes. Then we counted for each method the number of cases in which a significant value of mean *I*′ was obtained at the 10% level.

### Experimental data.

In the database MOUSE [21], samples of sequences from 345 populations were available. Samples containing fewer than 43 individuals were discarded. Because MOUSE contains a fairly small number of mtDNA samples from HGPs, mtDNA samples from one Asiatic HGP [22] and two African HGPs [23,24] were added to the sample. These three populations were chosen because their samples contained more than 43 sequenced individuals. mtDNA samples from region II of the D-loop were not included in the study since they were not available for most of the populations. The gene trees were built with PHYML using a gamma distribution for the mutation rate. The shape parameter of the gamma distribution was estimated by PHYML, and the number of substitution rate categories was fixed at four. The genealogies were rooted with one Pan paniscus sequence also available in MOUSE. Trees with fewer than four resolved nodes were discarded from the analysis.

## Abbreviations

FPPs: food-producer populations
HGPs: hunter–gatherer populations
